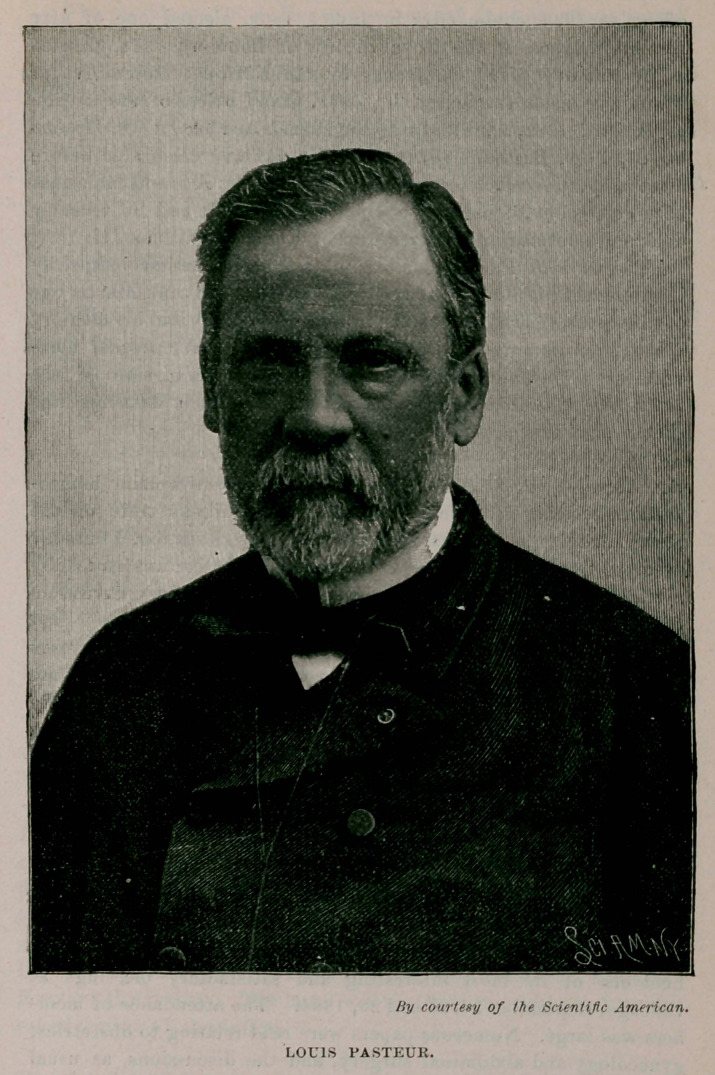# Louis Pasteur.—1822–1895

**Published:** 1895-11

**Authors:** 


### Obituary.

### LOUIS PASTEUR.—1822–1895.

If there were two opinions as to the merit of the work of Pasteur living, there is but one judgment of Pasteur dead—namely, that his memory will ever be cherished as one of the greatest men of his time.

Louis Pasteur was born at Dole, France, December 27, 1822, and died at Garches, near St. Cloud, in the environs of Paris, September 28, 1895. The immediate cause of his death was paralysis, which increased in severity during the last days of his life, culminating in a severe stroke the day before his death, after which he remained comatose until the end. Pasteur early developed a love for the study of chemistry, and entering the Ecole Normale at Paris he followed up his researches in this line of work. Afterward, at Sorbonne, he further pursued this study under the tuition of M. Dumas, whom he succeeded at the French Academy in 1882. Pasteur's first great work was accomplished in 1865–66, when he was called upon to investigate the silkworm plague that was destroying one of the greatest of French industries. He discovered that the cause of the plague was a parasite and that it could be arrested by destroying all the worms and eggs that were affected. He met the ridicule that this statement provoked, when he was told that the pest would still be propagated by spontaneous generation, by denying that there was any such thing and he proved the truth of his theories by checking the plague after the manner described.

The phenomenon of fermentation next attracted his attention. He alleged that it was caused by microörganisms and claimed that if all germs could be excluded fermentation would be impossible. He was again opposed by the same bigotry, but he demonstrated the truth of his propositions by showing that, at an altitude where the air was free from germs, no fermentation did or could occur.

Pasteur next began to investigate the diseases of men and animals, arguing that the contagious and infectious diseases were probably caused and sustained by the action of living organisms similar to the plague of the silkworm. It is well known to the profession of medicine that he soon sustained his theory and through his researches a large number of diseases have been brought under control.

Of late years Pasteur has turned his attention toward the cure of hydrophobia. While opinion has been and is yet divided as to the merits of his cure, there is yet an ever-increasing belief in its efficacy. Pasteur is entitled to the appellation of father of the germ theory of disease. Under the stimulus of his researches, diphtheria, cholera and hydrophobia are being stripped of their terrors, and it is believed that consumption will soon be under control.

In the following synopsis the principal events of Pasteur's life are given in chronological order: 1840, entered the university; 1843, pupil at the Ecole Normale; 1847, received the doctorate; 1848, professor of physics at Strasburg; 1854, dean of the faculty of sciences at Lille; 1857, assumed scientific direction of the Ecole Normale; 1863, professor of geology, physics and chemistry at the Ecole des Beaux-Arts; same year elected a member of the institute; 1856, awarded Rumford medal by the Royal Society of

*By courtesy of the Scientific American.*

LOUIS PASTEUR.

London; 1853, decorated with the Legion of Honor, promoted officer in 1863, commander in 1868; 1869, elected one of fifty foreign members of the Royal Society of London; 1874, granted a life annuity of 12,000 francs by the National Assembly for investigations on fermentation; 1878, grand officer of the Legion of Honor; 1882, admitted into the French Academy. On December 27, 1892, Pasteur's seventieth birthday was celebrated before a representative official assembly at Sorbonne. A mural tablet has been erected to his memory at the Ecole Normale and he recently declined a decoration tendered him by Emperor William III.

The work of Pasteur has been of a nature, whether judged by its present achievements or prospective results, to rank him as one of the greatest products of the nineteenth century and his memory will remain immortal in the realms of science. We present herewith an admirable portrait of Pasteur by permission of the *Scientific American* and we have obtained much data for this sketch from that paper of October 12, 1895.

## Figures and Tables

**Figure f1:**